# Automated Detection of Vaping-Related Tweets on Twitter During the 2019 EVALI Outbreak Using Machine Learning Classification

**DOI:** 10.3389/fdata.2022.770585

**Published:** 2022-02-10

**Authors:** Yang Ren, Dezhi Wu, Avineet Singh, Erin Kasson, Ming Huang, Patricia Cavazos-Rehg

**Affiliations:** ^1^Department of Computer Science and Engineering, University of South Carolina, Columbia, SC, United States; ^2^Department of Integrated Information Technology, University of South Carolina, Columbia, SC, United States; ^3^Department of Psychiatry, Washington University School of Medicine, St. Louis, MO, United States; ^4^Department of Artificial Intelligence and Informatics, Mayo Clinic, Rochester, MN, United States

**Keywords:** vaping, e-cigarette, Twitter, machine learning, deep learning, classification, detection, EVALI

## Abstract

There are increasingly strict regulations surrounding the purchase and use of combustible tobacco products (i.e., cigarettes); simultaneously, the use of other tobacco products, including e-cigarettes (i.e., vaping products), has dramatically increased. However, public attitudes toward vaping vary widely, and the health effects of vaping are still largely unknown. As a popular social media, Twitter contains rich information shared by users about their behaviors and experiences, including opinions on vaping. It is very challenging to identify vaping-related tweets to source useful information manually. In the current study, we proposed to develop a detection model to accurately identify vaping-related tweets using machine learning and deep learning methods. Specifically, we applied seven popular machine learning and deep learning algorithms, including Naïve Bayes, Support Vector Machine, Random Forest, XGBoost, Multilayer Perception, Transformer Neural Network, and stacking and voting ensemble models to build our customized classification model. We extracted a set of sample tweets during an outbreak of e-cigarette or vaping-related lung injury (EVALI) in 2019 and created an annotated corpus to train and evaluate these models. After comparing the performance of each model, we found that the stacking ensemble learning achieved the highest performance with an F1-score of 0.97. All models could achieve 0.90 or higher after tuning hyperparameters. The ensemble learning model has the best average performance. Our study findings provide informative guidelines and practical implications for the automated detection of themed social media data for public opinions and health surveillance purposes.

## Introduction

Recent data from the Center for Disease Control and Prevention (CDC) shows that over 8.1 million U.S. adults and 3.6 million youth use electronic cigarettes (i.e., e-cigarettes or vaping products) (Harold, 2020; Villarroel et al., 2020). Although companies that market vaping products state that vaping is less harmful than traditional cigarettes and can be used as a form of harm reduction, there are limited evaluations of the long-term health consequences that result from vaping, particularly among youth. To add to this relative uncertainty, an outbreak of acute consequences such as e-cigarette and vaping use-associated lung injury (EVALI) occurred in 2019 (Hajek, 2013; Goniewicz et al., 2014; Camenga and Tindle, 2018). This EVALI outbreak resulted in a total of 2,807 hospitalized cases with 68 confirmed deaths in 29 states and the District of Columbia, with a peak in September 2019, according to a CDC report (Centers for Disease Control and Prevention, 2020). The number of adults and youth vaping has evolved into a global public health crisis in the last decade, making it crucial to understand public perceptions and attitudes toward vaping and how these may relate to health behaviors and self-reported health outcomes.

Given the popularity of social media, many individuals use these platforms to connect with others and express themselves, including personal experiences with and opinions toward vaping. These social media platforms can serve as an excellent data source for collecting and mining vaping-related data. Twitter is one of the most popular social media applications, with 192 million daily active users reported at the end of 2020 (Digital Information World, 2021). Prior studies have successfully leveraged Twitter for health-related public surveillance in the areas of illicit drug use (Kazemi et al., 2017), mental health and wellbeing (Amir et al., 2019; Skaik and Inkpen, 2020), public health (Aiello et al., 2020), and other health-related topics (Jiang et al., 2018).

Despite its potential for public health surveillance, due to the large volume of tweets on Twitter, it would be highly challenging for mental and behavioral health providers to review all posts and replies to identify those which are vaping-related. Thus, the current study aims to develop a robust detection model to automatically capture vaping-related tweets and their associated user accounts by screening millions of tweets on Twitter. Our ultimate goal is to use this detection algorithm to effectively identify users at risk for adverse health outcomes due to vaping to reach out to those who may benefit from a vaping cessation intervention.

In this study, we propose to develop a high-efficiency detection model to automatically identify vaping-related tweets based on various machine learning and deep learning algorithms. To train and test the detection model to recognize vaping-related tweets, we created an annotated corpus as golden standard data consisting of vaping-related tweets as cases and other general non-vaping tweets as controls. The machine learning and deep learning algorithms include Naïve Bayes classifier, Support Vector Machine (SVM), Random Forest, XGBoost, Multilayer Perceptron (MLP), Transformer Neural Network, and stacking and voting structure-based ensemble learning methods (Das and Behera, 2017; Alzubi et al., 2018; Minaee et al., 2021). The annotated corpus and developed detection model may be helpful in future research to inform the customization of models for other research projects utilizing Twitter and other social media platforms. Compared with real-world settings, this study has its limitations, including the dataset's size, imbalanced test distribution, generalizability beyond training data, such as generating keywords, and evaluation bias from bot accounts. These limitations will be further handled in future work.

## Related Work

Social media platforms have become an essential part of public life. Previous literature has demonstrated that social media can be used to analyze public opinions on vaping and vaping-related behaviors, including their opinions between vaping and cannabis legalization (Adhikari et al., 2021), and perception of smoking behavior and emerging tobacco products (Myslín et al., 2013). Deploying predictive models with features extracted from Twitter, including tweet text, user profile information, geographic information, and sentiment, has been proven feasible in identifying vaping-related tweets in previous studies (Martinez et al., 2018). Extracted features can be considered as input variables in the standard machine learning algorithms, including SVM, Naïve Bayesian, and Random Forest, and have also been used successfully for topic analysis and detection (Aphinyanaphongs et al., 2016; Han and Kavuluru, 2016). Aphinyanaphongs et al. (2016) compared the performance of Naïve Bayes, Liblinear, Logistic Regression, and Random Forest classifiers to test the automatic detection of e-cigarette use (including e-cigarette use for smoking cessation) from tweet content (Aphinyanaphongs et al., 2016). Logistic Regression achieved the best performance (90% accuracy) for e-cigarette use detection, and Random Forest achieved the best performance (94% accuracy) for smoking cessation detection. For their Tweet sentiment analysis, positive sentiment indicates users' intention to use, the act of using, or sequel from use. Benson et al. (2020) investigated sentiment surrounding JUUL (i.e., an electronic nicotine delivery system) and vaping among youth and young adults by applying Logistic Regression, Naïve Bayes, and Random Forest for the detection of JUUL use and sentiment analysis. The Random Forest classifier achieved the best performance with 91% average detection accuracy among these classifiers. Moreover, due to their ability to learn complex non-linear functions, deep learning models have gained more popularity for detection tasks by feeding vectorized tweet contents as the model input (Visweswaran et al., 2020).

To design and justify our study, we reviewed relevant studies on vaping-related tweets analysis and cross-compared the scale of their dataset, setting, and performance of various machine learning and deep learning classifiers. The comparison results are presented in Table 1.

**Table 1:** Summary and cross-comparison of vaping-related twitter studies.

**Vaping-related Twitter studies**	**Subject**	**Scale of the dataset**	**Size of annotation**	**Classifier applied**	**Classifier setting (where applicable)**	**Best performance (accuracy)**
Adhikari et al. (2021)	Public opinions analysis about cannabis and JUUL on tweets	Dj:597,000 tweets from 2016 to 2018; Dc: 3.28M tweets from 2014 to 2018	500 tweets annotated from Dj, and 500 tweets annotated from Dc	Logistic Regression (LR), Support Vector Machine (SVM), LSTM-based Deep Neural Network (DNN)	Hyperparameters were tuned for each classifier	*Public opinions about cannabis and JUUL: micro*AUC *e-cigarette:* 0.93 *Cannabis:* 0.75
Myslín et al. (2013)	Tobacco-relevance tweets detection, positive & negative sentiment	7,362 tweets at 15-day intervals from December 2011 to July 2012 by keywords	Each of 7,362 tweets was manually classified	Naïve Bayes (NB), K-Nearest Neighbors (K-NN), SVM	Rainbow toolkit 10-fold cross-validation	*Tobacco-relevance tweets detection* NB: 0.77 K-NN: 0.73 SVM: 0.82
Martinez et al. (2018)	Public opinion about vaping investigates using sentiment analysis	973 tweets selected from 193,051 geocoded tweets within the U.S., and were collected between October 28, 2015 and February 6, 2016 by keywords	100 tweets were manually coded by two coders; Other tweets were single coded according to the codebook classifications	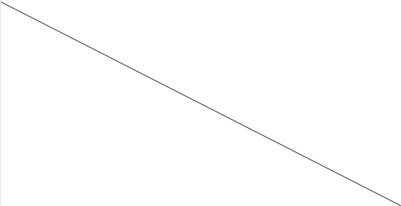	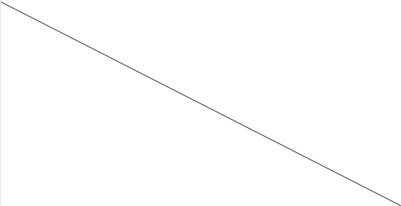	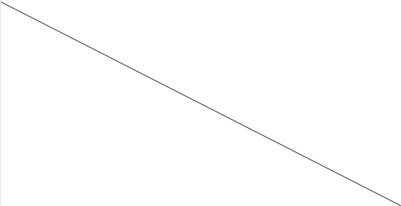
Aphinyanaphongs et al. (2016)	Vaping use and the detection of vaping use for smoking cessation tweets	13,146 tweets were selected from 228,145 tweets, collected between January 2010 and January 2015 by keywords	Each of 13,146 selected tweets was labeled by the classifiers	NB, SVM, LR, Random Forests (RF)	Parameters Settings: NB: Default SVM: Default LR: Auto search to optimize regularization parameter RF: Default	*Vaping use detection* NB: 0.82 SVM: 0.87 LR: 0.90 RF: 0.89 *Vaping Use for Smoking Cessation* NB: 0.60 SVM: 0.80 LR: 0.89 RF: 0.94
Han and Kavuluru (2016)	Marketing E- cigarette tweets detection and themes analysis	1,000 tweets were selected from 1,166,494 tweets obtained from April 2015 to June 2016 by keywords	Both authors independently annotated the 1,000 tweets	SVM, LR, Convolutional Neural Network (CNN)	Ten such models were run for each classifier on 10 different 80–20% train-test splits of the dataset	*E-cigarette tweets detection* SVM: 0.87 ± 0.01 LR: 0.88 ± 0.01 CNN: 0.88
Benson et al. (2020)	Adolescents and young adults for JUUL tweets detection and sentiment analysis	4,000 tweets were selected from 11,556 unique tweets containing a JUUL-related keyword	Manually annotated 4,000 tweets for JUUL-related themes of use and sentiment	LR, NB, RF	Grid search was applied to optimize hyperparameters 10-fold cross-validation	*Teen JUUL use tweets detection* LR: 0.94 NB: 0.78 RF: 0.99
Visweswaran et al. (2020)	The relevance and commercial Vaping-related tweets detection, and sentiment analysis	4,000 tweets were selected from 810,600 tweets extracted from August 2018 to October 2018 by vaping-related keywords	Manually annotated each of 4,000 tweets	LR, RF, SVM, NB, CNN, LSTM, LSTM-CNN, BiLSTM	Used default setting for the parameters in LR, RF, SVM. Tuned hyperparameters for CNN, LSTM, LSTM-CNN, BiLSTM	*Vaping tweets relevance detection* was based on vaping-related word vector: AUC LR: 0.84 RF: 0.95 SVM: 0.92 NB: 0.88 CNN:0.94 LSTM: 0.91 LSTM-CNN: 0.89 BiLSTM: 0.89

As shown in Table 1, Logistic Regression, Random Forest, SVM, and Naïve Bayes are the most used supervised machine learning classifiers for vaping-related Twitter studies, and deep neural networks (DNN) could also perform well in the tweet classification task. Hyperparameter tuning is necessary to improve the performance when building the classifiers. The appropriate splitting way for the training and testing set and validation method is also meaningful when building the classifiers. The typical approach of using 80% training set, 20% testing set, and cross-validation was applied in the previous studies. Since most of the previous research collected tweets in a long period (6 months or longer), their results cannot reflect the impact of specific events or changing public opinion tendencies.

In this study, we collected vaping-related and non-vaping-related tweets from July 2019 to September 2019. We only focused on these 3 months' peak period of EVALI outbreak in 2019 to avoid the ambiguity of long-period tweets analysis. Our clinical team also cross-checked these tweets to ensure no misclassified tweets in our dataset. We then built a detection model for vaping-related tweets by leveraging various machine learning and deep learning classifiers and cross-compared their detection performance metrics after tuning hyperparameters for each classifier. We also used ensemble learning models to compare the performance with baseline classifiers to identify the models with the highest performance.

## Methods

### Data Creation

#### Data Collection

In this project, we created an annotated corpus as a golden standard dataset to develop a detection model using machine learning and deep learning algorithms. We extracted Twitter data using the Twint Python package, an advanced open-source Twitter scraping tool that allows for scraping tweets from Twitter rather than using Twitter's official API (Pratama, 2020), limiting the extraction to 3,200 tweets with a 7-day history limit on each search. The Twint library tool provides a solution to bypass these limitations in data collection (Xavier and Souza, 2020).

The annotated corpus for the vaping-related tweet detection consisted of 1,506 vaping-related tweets and 1,464 general tweets not mentioning vaping. Each tweet included 10 or more words and was posted within the timeframe between July 2019 and September 2019. The tweet numbers in different months (July, August, and September) are shown in Table 2.

**Table 2:** Monthly distribution of tweets in the annotated corpus.

**Month in 2019**	**Vaping-related**	**Vaping not**	**Total tweets**
	**tweets**	**related tweets**	
July	498	495	993
August	499	502	1,001
September	509	467	976
Total	1,506	1,464	2,970

We included only tweets with 10 or more words to keep our dataset informative with more textual content to allow for analysis of semantic meaning and to further support the machine learning prediction. To create the annotated corpus, we collected and combined two separate sets of tweets: (1) a set of tweets was searched and extracted using vaping-related keywords, and (2) a set of tweets was collected through a random selection without using vaping-related keywords. The Twitter search keywords include ejuice, e-juice, eliquid, e-liquid, e-cigarette, e-cigs, electronic, vaporizer, vape, vaping, Pod-Mods, sub-ohm, MarkTen Elite, PAX Era, Eonsmoke, Eonsmoke, Vapor4Life, Puff Bar, njoy, and vuse. Then we need to identify the keywords selected tweets and randomly selected tweets related to vaping or not. Two individual clinical domain experts in mental health and substance use were recruited to label the combined dataset of selected vaping and non-vaping-related tweets. Each annotator manually reviewed and labeled vaping-related tweets as 1 and non-related tweets as 0. Inter-rater reliability was 93%. All discrepancies between the first 2 coders were resolved by a final consensus coder. The annotation results show that 254 keywords in the selected tweets were not related to vaping, and no vaping-related tweets were found in the randomly selected tweets. Then we added the 254 tweets to the set of randomly selected tweets as our control set. Finally, we obtained a new dataset with 1,506 tweets related to vaping and 1,464 tweets not related to vaping.

#### Data Preprocessing

We cleaned and processed the annotated corpus to prepare the tweet data for machine learning and deep learning algorithms by converting them into computational vectors (Brownlee, 2020). This data preprocessing allows us to conduct more efficient and accurate tweet data analyses to improve the overall performance of machine learning and deep learning models.

Following tweets preprocessing strategies used in previous literature (Irfan Alghani, 2020), our initial step was to convert the raw tweet text with noise into pure text. Unlike common texts, due to the 280-character limit of tweets and brevity of tweet writing style, users tend to add different types of non-text information when sending tweets that can be considered as “noise,” such as emojis, mentions (i.e., mentioning other Twitter user handles), hashtags symbol (#), and URLs. Although there are many mature models for non-textual data recognition, such as emoji recognition (LeCompte and Chen, 2017), these non-text pattern recognition models were not considered in this study as our focus was on the detection of vaping-related text within tweets.

More specifically, we converted tweet text to lowercase for all characters to avoid case sensitivity. We then removed unreadable Unicode characters, including emojis and other non-ASCII characters. In Twitter and other social media communities, users frequently use contractions in the limited text to communicate with others (Gómez-Adorno et al., 2016). We applied the contractions package to covert the contractions into original words to help with the data standardization and make the dataset ready for further process. Next, we focused on removing stop words, a type of word that has no specific meaning in the tweet text such as “is,” “be,” “are,” and “at,” etc. We used a stop word list from the NLTK library in previous research (Loper and Bird, 2002) to recognize and remove these words. After these cleaning steps, additional noises such as URLs, hashtags, mentions, punctuations, ticks with the next character, numbers, and over spaces remained for some tweets. To remove these remaining sources of noise from the dataset, we used regular expressions which contain the patterns to match and remove the target types of noise.

The next step was to tokenize the cleaned tweets into separate words and convert them into numerical vectors as machine learning and deep learning models inputs. We used the word tokenization function in the NLTK library to tokenize the cleaned tweets into separate words (Chakravarthy, 2020). Each word is called a token, which is used to analyze the words' sequence and to be vectorized further to develop our machine learning models. Before vectorizing the tokens for each tweet, we applied the word lemmatization function from the NLTK library to convert the words to their base forms. This step could reduce the size of word space to curb the sparsity of the data set and avoid model overfitting in further analysis (Camacho-Collados and Pilehvar, 2017). Both lemmatization and stemming approaches could lessen the word space (Jivani, 2011), but the result of lemmatization is the actual words which could provide more information when we look into the practical importance of the analysis results. Thus, we used the lemmatization approaches to convert the words into their base forms. We also applied the part-of-speech (POS) tag function provided by the NLTK library to assign a tag for each word in a specific context, such as noun, verb, adverb, adjective, determiner, etc. (Loper and Bird, 2002). These POS tags provide more evidence for the conversion process. Sequentially, we converted the textual data to numerical data before putting our dataset into the machine learning models as input. For this step, we applied Term Frequency-Inverse Document Frequency (TF-IDF), the most common method to transfer textual value to numerical value (Zhang et al., 2011). During the process of the TF-IDF, the tokens from the tokenizing process were converted to different feature indexes. The output features from TF-IDF were then fed into the machine learning models. In addition, for Transformer Neural Network, we used the word embedding technique as implemented in Keras (Gulli and Pal, 2017) to represent the cleaned tweets.

### Vaping-Related Tweets Detection Models

We built different vaping-related tweet detection models based on various machine learning algorithms, including Naive Bayes classifier, SVM, Random Forest, and XGBoost. We also deployed two deep learning models, a Multilayer Perceptron (MLP) model and a Transformer Neural Network model based on the Keras framework, to cross-compare the detection performance between machine learning classifiers and deep learning classifiers. We further tuned the setting of hyperparameters for each model based on our vaping detection dataset. Additionally, we combined several tree-based algorithms with building ensemble models and compared their performance with single tree-based machine learning classifiers and other machine learning models.

#### Machine Learning Classifier and Optimization

##### Naïve Bayes Classifier

The Naïve Bayesian algorithm is a supervised classification algorithm based on Bayes' theorem and assumes independence between features (Kiilu et al., 2018). It is widely used for text binary classification, sentiment analysis, and information filtering (Zhang and Li, 2007) due to its ability to handle small sample sizes with only a small amount of training data to estimate basic parameters.

This study applied the Gaussian Naive Bayes classifier as implemented in the Scikit-Learn (Pedregosa et al., 2011) Python package. There are two model parameters, Priors, and Var_smoothing, for the Gaussian Naive Bayes classifier as described in the Scikit-Learn official document. Priors indicate the prior probabilities of the classes. We kept the default setting to set the Priors as “None” because we did not input anything to the model as the prior experience. Var_smoothing is used for stability calculation by adding the portion of the largest variance of all features to their variances. The default value of Var_smoothing is 1e-9.

To optimize the model performance, we used the grid search algorithm to identify the optimal value of Var_smoothing. We set the searching range of the var_smoothing value from the default value of 1e-9 to 1e0 and generated 100 equally spaced candidate values within the range. We applied the 5-fold cross-validation to train and test the model for each candidate value and output the parameter value with the best model performance in each pair of training and testing sets.

##### Support Vector Machine (SVM)

SVM is a standard supervised machine learning algorithm for regression and classification problems, especially binary classification problems. The SVM algorithm finds a line or hyperplane in N-dimensional space that can best classify the data points. It is suitable for our binary text classification task because it is more effective in high dimensional space and performs well with small datasets (Liu et al., 2010).

In SVM, we optimized three major parameters to achieve the best model performance and get the optimal combination of parameters setting. The three major parameters include kernel, regularization parameter (C), and kernel coefficient parameter (Gamma). The kernel is a core function that transforms the input space from a lower dimension to a higher dimension in a non-linear fashion. The regularization parameter (C) is the penalty parameter that indicates the boundary of misclassification objects. The kernel coefficient parameter (Gamma) indicates the distance impact on the line of different classes separation. The parameters C and Gamma must be strictly positive. To find the optimal setting, we applied the grid search algorithm for three different kernels: sigmoid, polynomial, and radial basis function. The C's candidates are 0.1, 1, 10, 100, and 1,000. The choices of Gamma are 1, 0.1, 0.01, 0.001, and 0.0001 (Sunkad, 2016). As the hyperparameter tuning was processed in the Gaussian Naive Bayes classifier, we used grid search together with 5-fold cross-validation to find the optimal parameter setting with the best model performance in each pair of training and testing set.

##### Random Forest

Random Forests are among the most popular machine learning classification techniques, given their excellent accuracy, robustness, and ease of use (Roy and Larocque, 2012). The robustness of random forest is reflected in the capability of handling outliers. Based on the tree structural property, the outliers only impact the leaf node where the outliers belong to, but no impact on any other leaf node. Moreover, Random Forest classifiers effectively handle high dimensional, noisy data in text classification (Pranckevičius and Marcinkevičius, 2017). The Random Forest classifier with a bootstrap method generates different training sets, and the Random Forest algorithm constructs a decision tree for each training set. The features used in training each decision tree node are also randomly selected from the set of features. The benefit of using these random samples in both the training samples and the components of the feature vectors is its correction for the overfitting of decision trees, and thus all these decision trees form a robust Random Forest model. For classification problems, voting by multiple tree classifiers was used to determine the final classification result (Hastie et al., 2009).

To get the best performance of the random forest classifier, we tuned six important model parameters, which are n_estimators, max_features, max_depth, min_samples_split, min_samples_leaf, and bootstrap (Scornet, 2017). Compared with the Naive Bayes classifier and SVM, the model parameter space is vast, and it is costly to find the best combination of model parameters with the grid search algorithm. Thus, we used the random grid search algorithm to randomly sample the parameter combinations to approximate the best parameter setting (Bergstra and Bengio, 2012; Siji George and Sumathi, 2020). We set the range of the number of trees in the forest (n_estimators) from the default value 100–1,000 and generated 10 candidates with the searching range. We generated 10 candidates of the maximum depth of the tree (max_depth) for the searching range from 10 to 100. The min_samples_split is the minimum number of samples required to split an internal node, and its minimum value is 2. We chose 5 candidates from 2 to 10 for min_samples_split. The min_samples_leaf, the minimum number of samples required to be at a leaf node, has five candidates from 1 to 10 for searching. The bootstrap is a Boolean parameter that indicates whether the bootstrap samples are used when building trees. For the number of features to consider when looking for the best split (max_features), three different parameter types are included in the searching space: auto, sqrt, and log2. We used 5-fold cross-validation to evaluate the model performance and find the model with the best performance in each training and testing set combination.

##### XGBoost

XGBoost (Chen et al., 2015) is a supervised machine learning method for regression and classification tasks like the Random Forest classifier. Due to high execution speed, model performance, flexibility, and portability, XGBoost is popular in different data science competitions, like Kaggle, a data science community, and machine learning competition website (Chen and Guestrin, 2016).

XGBoost classifier has seven essential parameters, including n_estimators, learning_rate, max_depth, subsample, colsample_bytree, eta, and gamma for tuning the model performance (Budholiya et al., 2020; Ryu et al., 2020). Similar to the Random Forest classifier, we applied the random grid search method to optimize the seven essential parameters. We generated 10 candidates for the number of trees in a tree ensemble (n_estimators) from 100 to 1,000. We set 4 candidates (0.01, 0.1, 0.2, and 0.3) for the value of learning_rate. The candidates of the maximum depth of each tree (max_depth) were generated from 1 to 20 with a step size of 1. The subsample ratio of the training instances (subsample) prevents overfitting. We scanned the candidate value from 0.5 to 1 with a step size of 0.1. The parameter colsample_bytree indicates the subsample ratio of columns when constructing each tree, and the parameter candidates were generated incrementally by 0.1 from 0.1 to 1. The parameter eta is used to downsize the weights of features after each boosting step to prevent overfitting. The searching range of eta is from 1 to 2, and the interval between adjacent candidates is 0.1. Gamma is proportionate to the regularization level, and the candidate of gamma is scanned from 0 to 5 with an incremental step of 1. We deployed the 5-fold cross-validation to evaluate model performance and find the best performance in each training and testing set combination.

#### Deep Learning Classifier and Optimization

##### Multilayer Perceptron (MLP)

Multilayer Perceptron (MLP) is a deep learning neural network connecting multiple layers in a directed graph (Hastie et al., 2009). MLPs utilize non-linear activation functions on the hidden and output layers to distinguish data that is not linearly separable. MLP uses a supervised learning technique called backpropagation for training. After the feedforward calculation from the input layer to the output layer, the connection weights between layers are updated through backpropagation based on the amount of error in the output compared to the expected result for the supervised learning of MLP.

We applied the MLPClassifier as implemented in the Scikit-Learn Python package for the vaping detection task (Pedregosa et al., 2011). The MLP model consists of an input layer, multiple hidden layers, and an output layer. To build the MLP classifier with the best performance, we tuned five key model parameters, including the size of the hidden layers and the number of neurons in each layer (hidden_layer_sizes), the type of activation function (activation), the kind of solver for weight optimization (solver), the maximum number of iterations (max_iter), and the learning rate for weight updates (learning_rate) (Car et al., 2020; Weissbart, 2020). Given the size of the annotated corpus, we set the size of the hidden layers to be up to five layers, and the number of neurons in each layer from 100 to 500. The activation functions include tanh, relu, and logistic. There exist three different optimization solvers: sgd, adam, and lbfgs. The maximum iteration is scanned from 100 to 1,000 with an increment of 100. The types of learning rate include constant and adaptive. We applied the random grid search method and 5-fold cross-validation to find the optimal parameters with the best classifier performance.

##### Transformer Neural Network Classifier

We also built a Transformer Neural Network for the vaping-related tweets classification and compared the performance with other classifiers using Keras (Jakhar and Hooda, 2018). In this classifier, we first fed the token of words for each tweet into the embedding layer and then embedded the positional vector with the tokens and output this to the transformer layer. Multi-head attention was applied in the transformer layer to calculate scaled dot-production attention by the query, key, and values. The query, key, and value all came from the embedding input matrix. After normalizing the calculation results from multi-head attention, the transformer layer outputs one vector for each time step. It takes the mean value across all time steps to feedforward to the output of classification results (Vaswani et al., 2017; Apoorv, 2020).

To achieve the best performance of the Keras-based transformer neural network classifier and find the optimal parameter setting, we tuned the parameters using KerasTuner, which is a popular hyperparameter optimization framework under Keras (O'Malley et al., 2019; Rogachev and Melikhova, 2020). The tuned parameters include:

- The embedding size for each token
- The number of attention heads
- The hidden layer size in the feed forward network inside the transformer
- The dropout rates
- Activation function
- Optimizer function
- Learning rate

The range of the embedding size for each token was set from 32 to 512, and the interval between adjacent candidates was 32. We used the same searching strategy for the hidden layer size. The number of the attention heads is from 2 to 5. The candidates of dropout rate include 0.0, 0.01, 0.1, 0.2, and 0.3. The candidates of the activation function include rehu, tanh, and sigmoid. The types of optimizer functions include adam, sgd, and rmsprop. The candidates of the learning rate include 0.1, 0.01, and 0.001. We searched for the optimal parameter setting for the best model performance using the random grid search method in the KerasTuner package.

#### Ensemble Learning Classifier

**Figure 1:**
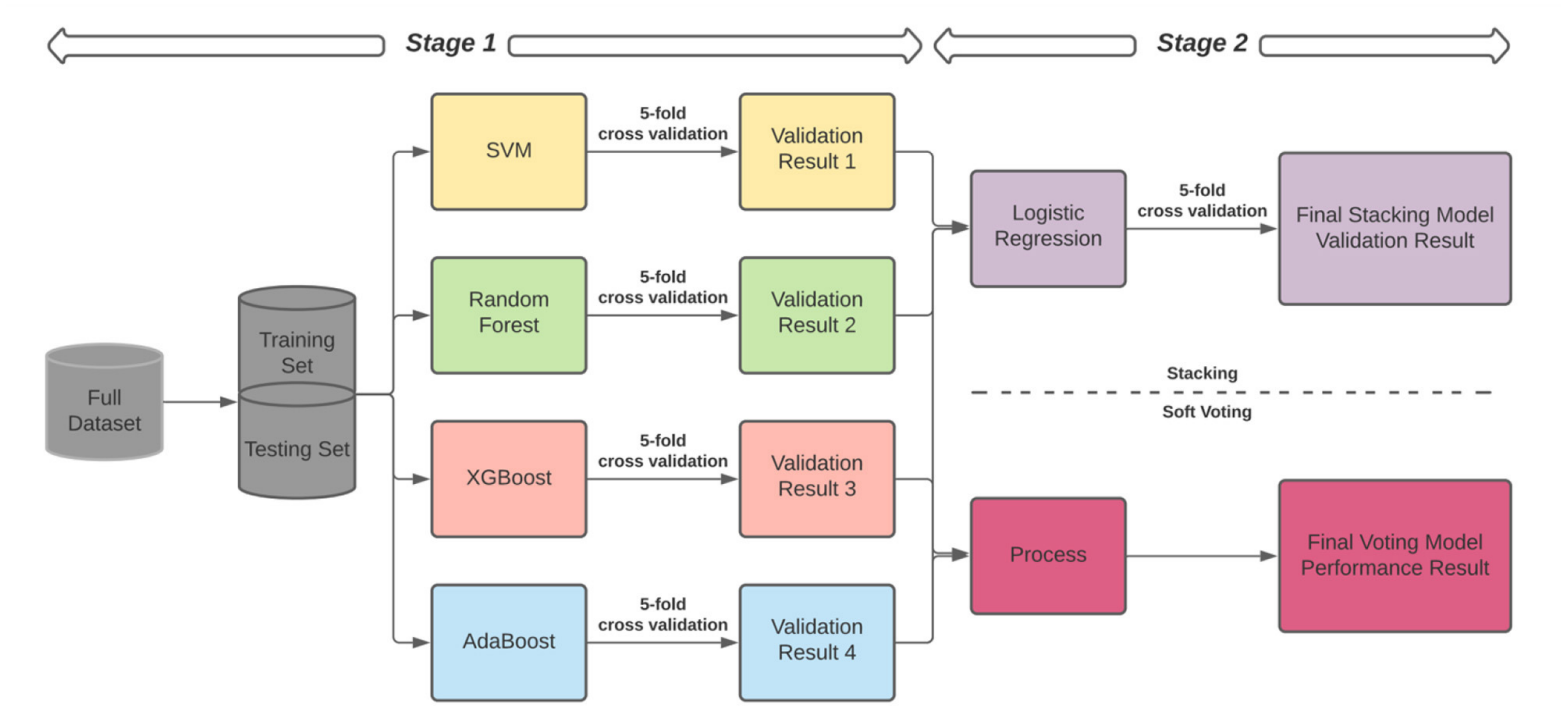
The structures of our ensemble learning model.

In addition to the single machine learning models described above, to obtain better performance and eliminate the biases from different single models, we applied the ensemble learning method to combine the base machine learning models to get a better, more comprehensive, and strongly supervised model (Xiao et al., 2018). To establish the final ensemble model, we incorporated two common structures, including stacking and soft voting to compare the performance (Figure 1). The two most popular ensemble methods, bagging and boosting, have different preferences. The bagging method is suitable for eliminating the overfitting problem but could increase the bias, and the boosting method could reduce the bias but may lead to the overfitting issue. To get an ensemble model with better performance and avoid the disadvantages of using single bagging and boosting model, we selected four different base models: a typical linear model for classification—SVM, a most common model with the bagging method—Random Forest, and two standard models with the boosting method—XGBoost and AdaBoost. In the ensemble model, we applied stacking and soft voting strategies to evaluate the results from these four base models.

As shown in Figure 1, there were two training stages in the stacking structure. In Stage 1, we trained a set of base machine learning models and generated prediction results by a 5-fold cross-validation process by splitting the training set into five subsets (Rodriguez et al., 2009). We used four subsets to train all base machine learning classifiers in the ensemble model, assigned the remaining subset as the testing set, and evaluated the model performance. Next, we used a different subset as the testing set to run the same process until all groups were applied as a testing set once.

Based on the stacking algorithm, in Stage 2, we constructed a new data set based on the output from the single classifiers in Stage 1 (Figure 1). The output predicted labels of the classifiers in Stage 1 are regarded as the new input features, and the labels in the original dataset are the labels in the new data set (Odegua, 2019). Then we used Logistic Regression to train the final stacking model based on the new dataset and obtained the final performance. Separately in Stage 2 we also applied the soft voting classifier to the new training set generated in Stage 1 to calculate the probabilities of each class from different base models. These probabilities were averaged with equal weights in this step. We selected the highest weighted and averaged probability to determine the final voting result. The final output is the method with the highest performance based on the testing set among stacking and voting.

### Experiments and Evaluation

This study builds and optimizes different machine learning and deep learning classifiers based on the annotated dataset for the vaping detection task. We tuned the model parameters and hyperparameters and evaluated the model performance using accuracy, precision, recall, and F1-score.


$$\begin{array}{l} \text{Accuracy}=\;\frac{\text{True Negative + True Positive}}{\text{True}\;\text{Negative}\;+\;\text{True}\;\text{Positive}\;+}\\ \;\text{False}\;\text{Negative}+\text{False}\;\text{Positive}\\ \;\text{Precision}\text{ = }\frac{\text{True Positive}}{\text{True Positive + False Positive}}\;\\ \;\mathrm{Recall}\text{ = }\frac{\text{True Positive}}{\text{True Positive + False Negative}}\\ \text{ F1-}\text{score}\text{ = }\frac{\text{2 }*\;(\text{Precision }*\text{ Recall})}{\text{Precision}+\text{Recall}} \end{array}$$

True-positive denotes the number of positive classes correctly predicted by the model. False-positive means the number of the positive class incorrectly predicted by the model. True-negative refers to as the number of the negative classes correctly predicted by the model. False-negative is the number of the negative classes that incorrectly predicted the model.

In addition, we experimented with two different spitting strategies of the training and testing sets to evaluate the classifiers based on the *month-based* and *percentage-based* methods. For the month-based split method, we used two out of 3 months' data as the training set and another month's data as the testing set, generating three different training-testing combinations. For the percentage-based method, we split the dataset by six different percentages: 50% training and 50% testing, 60% training and 40% testing, 70% training and 30% testing, 80% training and 20% testing, 90% training, and 10% testing. The similar language and utterances from the same user's tweets in the training and testing sets could bring evaluation bias. To avoid biased evaluations, we checked our dataset by the user ID and tweet ID with the tweet content to ensure no tweets from the same users or retweets by other users overlapped between the training and testing sets when we split our dataset.

## Results

This section presents our experimental and evaluation results based on each classifier's training and testing combination and cross-comparison.

### Model Performance

Table 3 shows the best performance achieved by each classifier for all different training-testing combinations and hyperparameters settings. As shown in Table 3, the stacking ensemble method achieved the highest F1-Score 0.97 based on all different training and testing set combinations (Tables A13, A14 in Appendix). Random Forest and Transformer classifiers achieved the second high F1-Score 0.96 (Tables A5, A6, A11, A12 in Appendix). The highest F1-Score of Naïve Bayes, SVM is 0.95. The best F1-Score for the MLP classifier is 0.94, and XGBoost got 0.92. The detail of the results for each classifier is shown in Tables A1, A14 in Appendix.

**Table 3:** Best performance (F1-score) achieved for each classifier.

**Classifier**	**Accuracy**	**Precision**	**Recall**	**F1-Score**	**Training set**	**Testing set**
Naïve Bayes	0.94	0.93	0.96	0.95	90%	10%
SVM	0.95	0.98	0.92	0.95	7, 8 (Month)	9 (Month)
	0.95	0.96	0.94	0.95	90%	10%
	0.94	0.96	0.95	0.95	70%	30%
Random Forest	0.95/0.96	0.96/0.95/0.94/0.93	0.97/0.96	0.96	90%/70%/ 60%/50% 7, 8 (Month) 7, 9 (Month)	10%/30% 40%/50% 9 (Month) 8 (Month)
XGBoost	0.91/0.92	0.94/0.93/0.91	0.91/0.92	0.92	All training sets are based on various month and percentage combinations except: 7, 8 (Month)	All testing sets are based on various month and percentage combinations except: 9 (Month)
Ensemble - Stacking	0.97	0.97	0.97	0.97	All training sets are based on various month and percentage combinations	All testing sets are based on various month and percentage combinations
MLP	0.94	0.94	0.94	0.94	7, 9 (Month)	8 (Month)
	0.94	0.94	0.94	0.94	50%	50%
Transformer	0.96	0.96	0.96	0.96	7, 8 (Month)	9 (Month)

### Temporal Experiment

**Figure 2:**
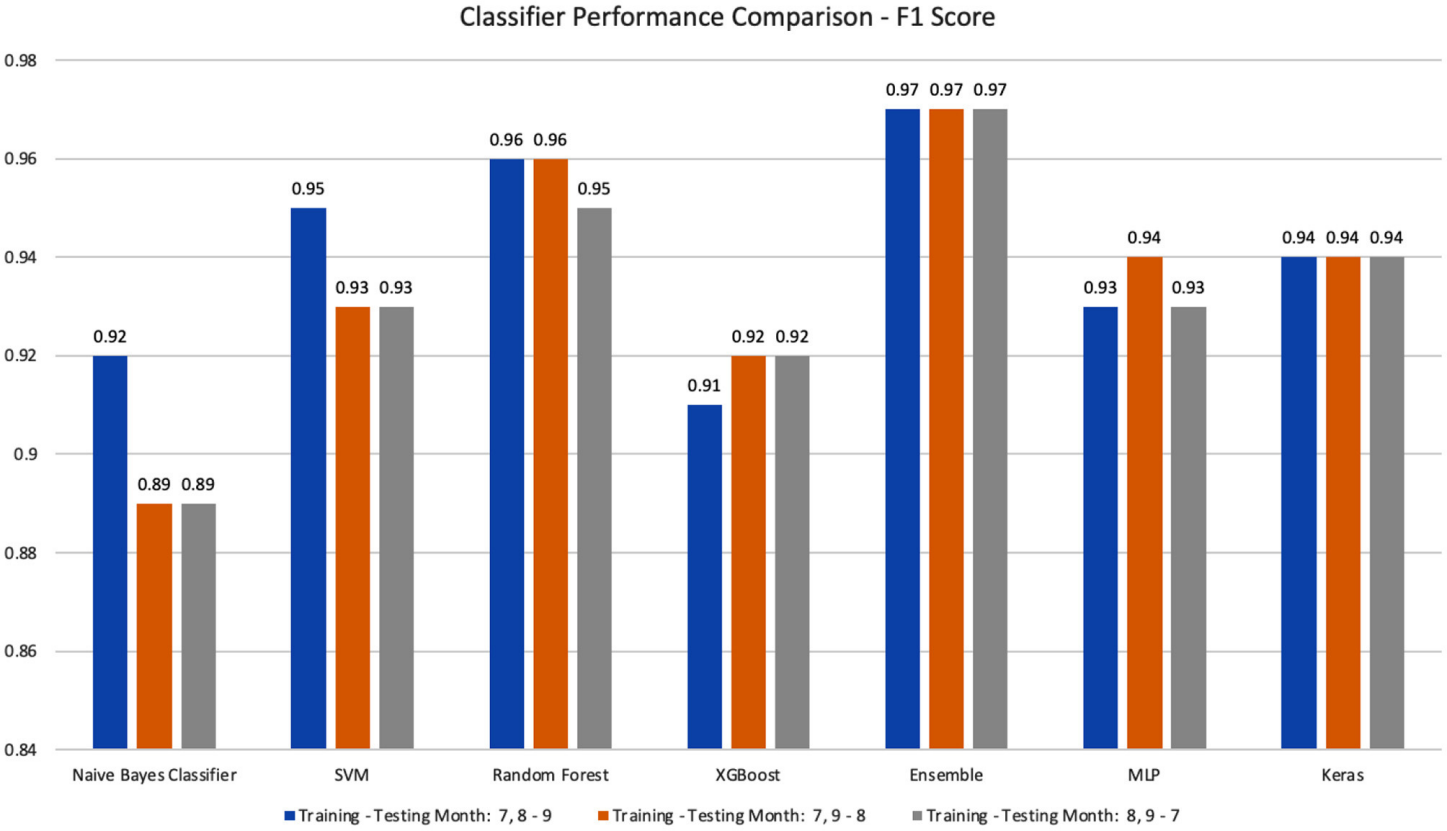
The performance comparison for all classifiers based on different month-based training-testing combinations.

We performed temporal experiments based on different combinations of month-based training and testing datasets (i.e., Training-testing Months of 7, 8–9, 7, 9–8, and 8, 9–7). Figure 2 shows the best prediction accuracy achieved in each month-based training and testing combination. All classifiers can accurately classify vaping-related tweets with an F1-Score of 0.92 or better. The best detection model is stacking ensemble (0.97, 0.97, 0.97), and the second-best model is Random Forest (0.96, 0.96, 0.95) for three different month-based training-testing settings. After cross comparing the different month-based training and testing set combinations, we found that all classifiers achieved 0.91 or higher F1-Score except Naïve Bayes. The stacking ensemble and transformer classifiers have the most stable performance for all three training and testing set combinations. The detailed results of their testing accuracy, precision, recall, F1-score, and the optimal parameter value are shown in Tables A1–A14 in Appendix.

### Testing Size Experiment

**Figure 3:**
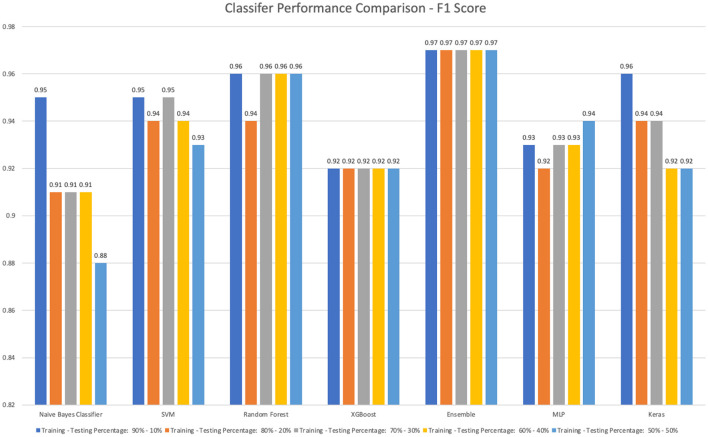
The performance comparison for all classifiers based on percentage-based different training-testing combinations.

We also experimented with a percentage-based split of training and testing sets (i.e., different combinations of Training-testing percentages: 90–10%, 80–20%, 70–30%, 60–40%, and 50–50%). Figure 3 shows the best prediction accuracy achieved in each percentage-based training and testing combination. The results are based on the month-based training and testing combinations, except the Naïve Bayes classifier, and all other classifiers can accurately classify vaping-related tweets 0.91 or a better F1-Score. The top 3 detection models are stacking ensemble (0.97, 0.97, 0.97, 0.97, 0.97), Random Forest (0.96, 0.94, 0.96, 0.96, 0.96), and SVM (0.95, 0.94, 0.95, 0.94, 0.93) for different percentage-based training and testing settings. The stacking ensemble classifier is still the most stable model for all training-testing set combinations. The detailed results of testing accuracy, precision, recall, F1-score, and the optimal parameter value are shown in Tables A1–A14 in Appendix.

### Feature Analysis

**Figure 4:**
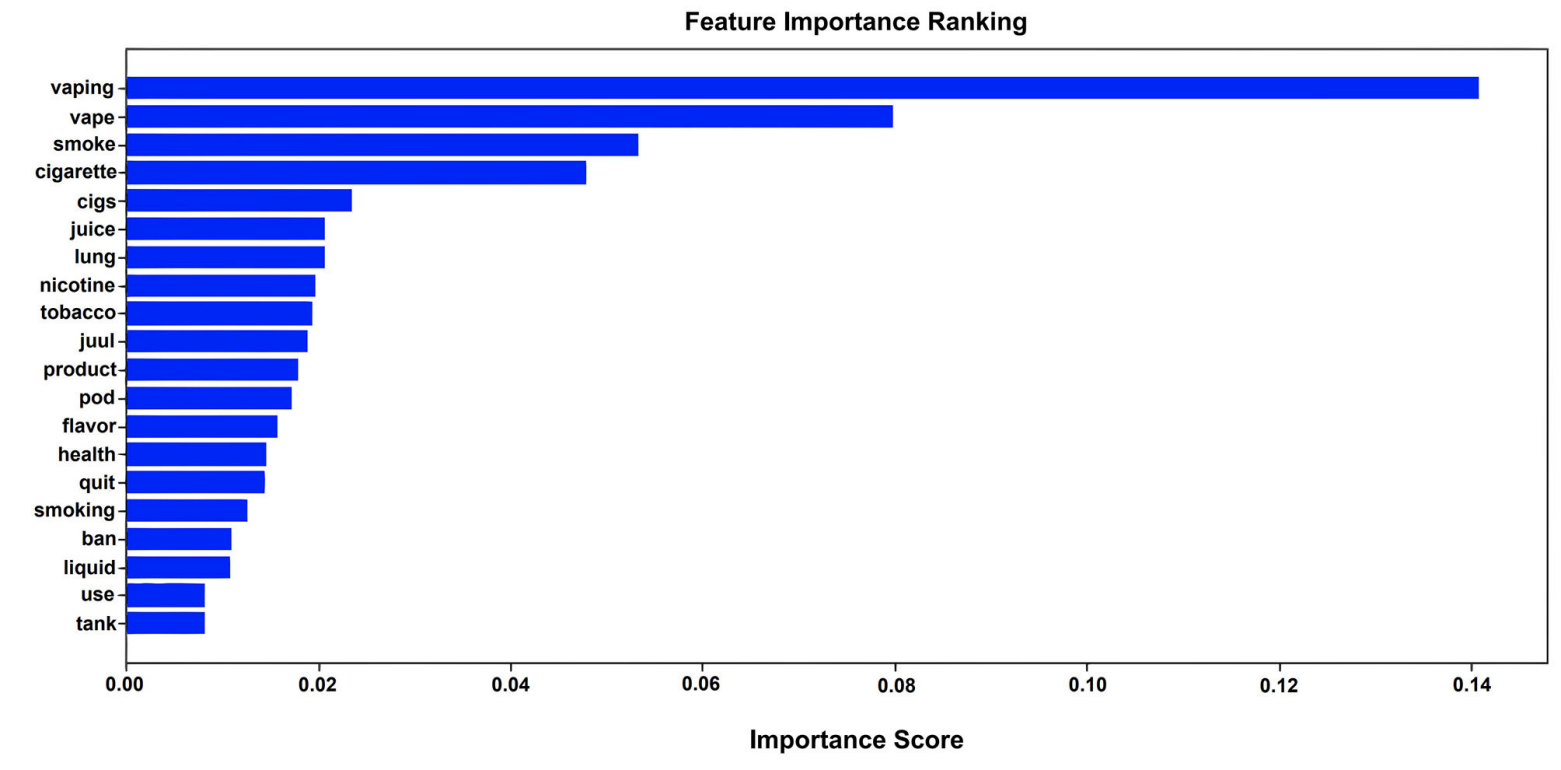
Top 20 important features based on random forest classifier.

To further understand the characteristics of vaping-related tweets, we applied the Random Forest classifier to analyze feature importance. Then top 20 important features were identified for the detection of vaping-related tweets, as shown in Figure 4, in which the y-axis represents the feature names, and the x-axle indicates the importance score for each feature, calculated through Gini importance (Qi, 2012) for each node on each decision tree and an average overall the trees based on the sum of all feature importance values. The final importance score was normalized into the scale 0 and 1. The higher value indicates the more important feature (Breiman, 2001; Ronaghan, 2018).

We grouped the most important words into two major categories, including *smoking-related* and *health-outcome-related* words based on their literal meaning, without considering the context of tweets, and thus the words from these four categories could occur in the same tweets. In detail, these two groups of words include (1) *Smoking-related common words*: vaping, vape, smoke, cigarette, cigs, nicotine, tobacco, smoking. (2) *The health-outcome-related words*: lung, quit, use. (3) *Vaping product-related words*: juice, juul, product, pod, flavor, liquid, tank. (4). *Policy-related word*: ban. The presence of these 20 important words indicate that more vaping-related tweets mentioned vaping or smoking health-related outcomes during the outbreak of EVALI. Furthermore, there are 15 out of 20 most important words about smoking and vaping products, which indicate that the topic of vaping products is also popular in the Twitter community. Moreover, a certain number of tweets are focused on banning vaping.

## Evaluation

In this section, to validate the results based on the input vectors from TF-IDF, we applied word2vec for the new dataset. Word2vec is a popular method for learning word embeddings based on a two-layer neural network to convert the text data into a set of vectors (Mikolov et al., 2013). Unlike TF-IDF, word2vec could consider more context when processing each word (Kurnia et al., 2020). We applied word2vec with a skip-gram training algorithm given by the Gensim library (Rehurek and Sojka, 2011). We applied the results from word2vec as the input to the classification models except for Keras, which has a similar embedding layer for text vectorization.

Table 4 shows the best performance achieved for each classifier among all different training-testing combinations and hyperparameters settings based on the new dataset—the highest F1-Score 0.97 achieved by the stacking ensemble model. SVM and MLP achieved second high F1-Score 0.95. All models still achieved 0.9 or higher F1- Score for all training-testing combinations, excepting Naïve Bayes, which got lower than 0.9 F1-Score for 6 out of 8 training-testing combinations. SVM and MLP achieved their best performance in the same training-testing combinations and hyperparameters settings as the original results. These results provide evidence to show our classification models' generalizability and the validation of previous results. The detail of the results for each classifier is shown in Tables A15, A16 in Appendix.

**Table 4:** Best performance achieved for each classifier—evaluation dataset.

**Classifier**	**Accuracy**	**Precision**	**Recall**	**F1-score**	**Training set**	**Testing set**
Naïve Bayes	0.90	0.95	0.86	0.90	7, 8 (Month)	9 (Month)
	0.90	0.92	0.88	0.90	8, 9 (Month)	7 (Month)
SVM	0.95	0.96	0.94	0.95	7, 8 (Month)	9 (Month)
	0.95	0.96	0.94	0.95	90%	10%
Random Forest	0.94	0.94	0.94	0.94	7, 8 (Month)	9 (Month)
	0.94	0.94	0.93	0.94	8, 9 (Month)	7 (Month)
XGBoost	0.94	0.94	0.94	0.94	7, 8 (Month)	9 (Month)
Ensemble—stacking	0.95	0.97	0.97	0.97	7, 9 (Month) 80% 70%	8 (Month) 20% 30%
MLP	0.95	0.95	0.95	0.95	7, 8 (Month)	9 (Month)
	0.95	0.95	0.95	0.95	90%	10%

## Discussion

This study extracted vaping-related tweets from the Twitter platform and created an annotated corpus for developing a detection model. We built different detection models based on TF-IDF, word embedding techniques and popular machine learning and deep learning algorithms. Model performance comparisons demonstrated that all machine learning and deep learning models for our small sample of textual data with high dimensions could achieve F1-Score>0.91. The ensemble learning classifier (stacking) achieved the best average detection performance. The stacking approach, which created the first-stage features by the single classifiers and transformed the data into another feature space to predict the actual target in the second stage, could slightly improve the vaping detection performance among other machine learning and deep learning classifiers that we evaluated.

After comparing our vaping-related tweets detection performance with the classifier results reported in previous studies (Table 1), we found that the Random Forest, SVM, and Transformer classifiers have constantly achieved better performance than the Naïve Bayes classifier. This finding is consistent in our study that Random Forest, SVM, and Transformer perform better in executing text content classification tasks than the Naïve Bayes. Unlike previous studies that only built the classifiers on the same training and testing combination, we developed our classifiers based on six different training and testing combinations and examined the optimal hyperparameter setting for each classifier. We also evaluated our classification models based on the word2vec vectorization method. The classification models also achieved high accuracy from the new dataset and supported the initial results—the average performance of Naïve Bayes is not as good as other classifiers for the vaping detection task. Furthermore, the stacking ensemble model could perform better than other models.

Recently, the pre-trained deep learning methods have shown promising results in natural language processing tasks, including text classification, so we plan to explore these pre-trained models and their variants to detect vaping-related tweets in the future.

The current study results effectively support the feasibility and validity of using detection models based on ensemble classifier with stacking method to identify vaping-related tweets on Twitter. Such approaches to detection and combined with additional analyses, have the potential to screen and mine millions of tweets to identify individuals who are communicating and networking about vaping on social media sites and to reach out to those who may be at risk for adverse health outcomes due to vaping and could benefit from direct connection to cessation support and related intervention programs.

## Limitation

The first limitation of this study is the size and distribution of our dataset. In the current dataset, we have 1,506 vaping-related tweets and 1,464 vaping-not-related tweets. In the real-world situation, the vaping topic is a small part of all tweets in the Twitter community. Still, we did not use many unrelated vaping tweets instead of a sample to form an imbalanced dataset to evaluate the vaping detection classifiers. Since clinic experts need to cross-check all collected tweets to determine whether the tweets are related to vaping or not, we cannot overextend the size of our data due to the time-consuming human manual check process and resource limitations.

The second limitation of this study is its generalizability. We collected the vaping-related tweets based on a set of the keywords generated by our clinic team, given that the keyword search is a standard method when we search specific content on the Internet. The additional human check process has helped avoid the impact of keyword filtering on recall and precision. The limitation of keyword search is that irrelevant content might be included since the keyword search cannot accurately identify the words' semantic meaning in different contexts, and thus may cause negative effects on recall and precision. In our study, our clinical team checked each tweet from keywords selection when they annotated the tweets to ensure they were all related to the vaping topic that we focused on in this study. As such, there might be a lack of generalizability to expand this keyword set, which is not the current scope of this work.

Another limitation is the bias in our current dataset. The tweets possibly generated from the bot could be included in the dataset. At the current stage of this study, we did not apply any filter to remove these tweets since our primary target of this study is to detect whether the tweets are related to vaping or not.

## Data Availability Statement

The datasets presented in this article are not readily available because there are some potentially identifiable data. Requests to access the datasets should be directed to dezhiwu@cec.sc.edu.

## Author Contributions

YR conducted all machine learning classification tasks and drafted the initial results. DW conceptualized the manuscript with writing, editing, and revised the manuscript. AS extracted Twitter sample data, conducted data cleaning, and preliminary analysis. EK and PC-R guided human coding, clinical implications, and edited the paper. MH designed the research study, conceived the methods, discussed results, and revised the manuscript. All authors read the current manuscript and approved the submitted version.

## Funding

The authors would like to acknowledge the funding support provided by the grants of University of South Carolina (USC), Columbia, Unites States (No. 80002838), and partial support from the USC Big Data Health Science Center, a USC excellence initiative program (No. BDHSC-2021-14), National Institutes of Health (NIH) (No. K02 DA043657 AWARD), and Mayo Clinic Center for Clinical and Translational Science (No. UL1TR02377).

## Author Disclaimer

The content is solely the authors' responsibility and does not necessarily represent the official views of the funding agencies.

## Conflict of Interest

The authors declare that the research was conducted in the absence of any commercial or financial relationships that could be construed as a potential conflict of interest.

## Publisher's Note

All claims expressed in this article are solely those of the authors and do not necessarily represent those of their affiliated organizations, or those of the publisher, the editors and the reviewers. Any product that may be evaluated in this article, or claim that may be made by its manufacturer, is not guaranteed or endorsed by the publisher.
