# Supplementary material for: Automated Detection of Vaping-Related Tweets on Twitter During the 2019 EVALI Outbreak Using Machine Learning Classification
Source: Front Big Data. 2022 Feb 10;5:770585. doi: 10.3389/fdata.2022.770585 (PMC8866955; doi:10.3389/fdata.2022.770585)
Supplement: Supplementary file 1 [file Table_1.docx]

**Appendix:**

| **Training Set (Month)** | **Testing Set (Month)** | **Precision** | **Recall** | **F1-Score** | **Accuracy** | **Var_Smoothing Value (Optimal)** |
| --- | --- | --- | --- | --- | --- | --- |
| 7, 8 | 9 | 0.92 | 0.93 | 0.92 | 0.92 | 1.0 |
| 7, 9 | 8 | 0.87 | 0.91 | 0.89 | 0.89 | 1.52e-9 |
| 8, 9 | 7 | 0.86 | 0.94 | 0.90 | 0.89 | 2.85e-8 |

Table A1. Naive Bayes Classifier Final Results – Month

| **Training Set (Percentage)** | **Testing Set (Percentage)** | **Precision** | **Recall** | **F1-Score** | **Accuracy** | **Var_Smoothing Value (optimal)** |
| --- | --- | --- | --- | --- | --- | --- |
| 90% | 10% | 0.93 | 0.96 | 0.95 | 0.94 | 5.34e-9 |
| 80% | 20% | 0.89 | 0.95 | 0.92 | 0.91 | 2.85e-9 |
| 70% | 30% | 0.89 | 0.94 | 0.92 | 0.91 | 1e-9 |
| 60% | 40% | 0.89 | 0.94 | 0.91 | 0.91 | 2.85e-9 |
| 50% | 50% | 0.83 | 0.88 | 0.88 | 0.88 | 1.0 |

Table A2. Naive Bayes Classifier Final Results – Percentage

| **Training Set (Month)** | **Testing Set (Month)** | **Precision** | **Recall** | **F1-Score** | **Accuracy** | **Kernel** | **C** | **Gamma** |
| --- | --- | --- | --- | --- | --- | --- | --- | --- |
| 7, 8 | 9 | 0.98 | 0.92 | 0.95 | 0.95 | Sigmoid | 1 | 1 |
| 7, 9 | 8 | 0.96 | 0.90 | 0.93 | 0.93 | Sigmoid | 1000 | 0.001 |
| 8, 9 | 7 | 0.94 | 0.92 | 0.93 | 0.93 | Sigmoid | 1 | 1 |

Table A3. SVM Final Results – Month

| **Training Set (Percentage)** | **Testing Set (Percentage)** | **Precision** | **Recall** | **F1-Score** | **Accuracy** | **Kernel** | **C** | **Gamma** |
| --- | --- | --- | --- | --- | --- | --- | --- | --- |
| 90% | 10% | 0.96 | 0.94 | 0.95 | 0.95 | Sigmoid | 10 | 0.1 |
| 80% | 20% | 0.94 | 0.93 | 0.94 | 0.94 | Sigmoid | 10 | 0.1 |
| 70% | 30% | 0.96 | 0.95 | 0.95 | 0.94 | Sigmoid | 10 | 0.1 |
| 60% | 40% | 0.97 | 0.90 | 0.94 | 0.94 | Sigmoid | 10 | 0.1 |
| 50% | 50% | 0.97 | 0.99 | 0.93 | 0.93 | Sigmoid | 10 | 1 |

Table A4. SVM Final Results – Percentage

| **Training Set (Month)** | **Testing Set (Month)** | **Precision** | **Recall** | **F1-Score** | **Accuracy** | **Parameter Setting (Optimal)** |
| --- | --- | --- | --- | --- | --- | --- |
| 7, 8 | 9 | 0.96 | 0.97 | 0.96 | 0.96 | n_estimators: 700,  min_samples_split: 6,  min_samples_leaf: 1,  max_features: auto,  max_depth: 100,  bootstrap: False |
| 7, 9 | 8 | 0.96 | 0.96 | 0.96 | 0.96 | n_estimators: 500,  min_samples_split: 8,  min_samples_leaf: 1,  max_features: auto,  max_depth: 70,  bootstrap: False |
| 8, 9 | 7 | 0.94 | 0.96 | 0.95 | 0.95 | n_estimators: 300,  min_samples_split: 6,  min_samples_leaf: 1,  max_features: auto,  max_depth: 70,  bootstrap: False |

Table A5. Random Forest Final Results – Month

| **Training Set (Percentage)** | **Testing Set (Percentage)** | **Precision** | **Recall** | **F1-Score** | **Accuracy** | **Parameter Setting**  **(Optimal)** |
| --- | --- | --- | --- | --- | --- | --- |
| 90% | 10% | 0.94 | 0.97 | 0.96 | 0.95 | n_estimators: 300,  min_samples_split: 4,  min_samples_leaf: 1,  max_features: auto,  max_depth: 60,  bootstrap: False |
| 80% | 20% | 0.93 | 0.96 | 0.94 | 0.94 | n_estimators: 300,  min_samples_split: 2,  min_samples_leaf: 1,  max_features: auto,  max_depth: 60,  bootstrap: False |
| 70% | 30% | 0.95 | 0.96 | 0.96 | 0.95 | n_estimators: 200,  min_samples_split: 2,  min_samples_leaf: 1,  max_features: sqrt,  max_depth: 60,  bootstrap: False |
| 60% | 40% | 0.95 | 0.97 | 0.96 | 0.96 | n_estimators: 300,  min_samples_split: 4,  min_samples_leaf: 1,  max_features: sqrt,  max_depth: 60,  bootstrap: False |
| 50% | 50% | 0.94 | 0.97 | 0.96 | 0.95 | n_estimators: 500,  min_samples_split: 8,  min_samples_leaf: 1,  max_features: sqrt,  max_depth: 60,  bootstrap: False |

Table A6. Random Forest Final Results – Percentage

| **Training Set (Month)** | **Testing Set (Month)** | **Precision** | **Recall** | **F1-Score** | **Accuracy** | **Parameter Setting**  **(Optimal)** |
| --- | --- | --- | --- | --- | --- | --- |
| 7, 8 | 9 | 0.93 | 0.91 | 0.91 | 0.91 | subsample: 0.5,  n_estimators: 700,  max_depth: 1,  learning_rate: 0.1,  gamma: 0,  eta: 1.1,  colsample_bytree: 0.4 |
| 7, 9 | 8 | 0.93 | 0.91 | 0.91 | 0.91 | subsample: 0.6,  n_estimators: 800,  max_depth: 1,  learning_rate: 0.1,  gamma: 0,  eta: 1.7,  colsample_bytree: 0.5 |
| 8, 9 | 7 | 0.94 | 0.92 | 0.92 | 0.92 | subsample: 0.6,  n_estimators: 700,  max_depth: 1,  learning_rate: 0.1,  gamma: 0,  eta: 1.6,  colsample_bytree: 0.6 |

Table A7. XGBoost Final Results – Month

| **Training Set (Percentage)** | **Testing Set (Percentage)** | **Precision** | **Recall** | **F1-Score** | **Accuracy** | **Parameter Setting**  **(Optimal)** |
| --- | --- | --- | --- | --- | --- | --- |
| 90% | 10% | 0.94 | 0.91 | 0.92 | 0.91 | subsample: 0.9,  n_estimators: 200,  max_depth: 1,  learning_rate: 0.3,  gamma: 0,  eta: 1.4,  colsample_bytree: 0.5 |
| 80% | 20% | 0.93 | 0.92 | 0.92 | 0.92 | subsample: 0.9,  n_estimators: 800,  max_depth: 1,  learning_rate: 0.1,  gamma: 0,  eta: 1.0,  colsample_bytree: 0.6 |
| 70% | 30% | 0.93 | 0.92 | 0.92 | 0.92 | subsample: 0.5,  n_estimators: 600,  max_depth: 1,  learning_rate: 0.1,  gamma: 0,  eta: 1.2,  colsample_bytree: 0.4 |
| 60% | 40% | 0.93 | 0.91 | 0.92 | 0.91 | subsample: 0.7,  n_estimators: 600,  max_depth: 1,  learning_rate: 0.1,  gamma: 0,  eta: 1.0,  colsample_bytree: 0.6 |
| 50% | 50% | 0.93 | 0.91 | 0.92 | 0.91 | subsample: 0.7,  n_estimators: 600,  max_depth: 1,  learning_rate: 0.1,  gamma: 0,  eta: 1.0,  colsample_bytree: 0.4 |

Table A8. XGBoost Final Results – Percentage

| **Training Set (Month)** | **Testing Set (Month)** | **Precision** | **Recall** | **F1-Score** | **Accuracy** | **Parameter Setting**  **(Optimal)** |
| --- | --- | --- | --- | --- | --- | --- |
| 7, 8 | 9 | 0.94 | 0.93 | 0.93 | 0.93 | solver: lbfgs,  max_iter: 500,  learning_rate: constant,  hidden_layer: (100, 100),  activation: logistic |
| 7, 9 | 8 | 0.94 | 0.94 | 0.94 | 0.94 | solver: sgd,  max_iter: 700,  learning_rate: constant,  hidden_layer:  (500, 250, 500),  activation: tanh |
| 8, 9 | 7 | 0.93 | 0.93 | 0.93 | 0.93 | solver: lbfgs,  max_iter: 400,  learning_rate: adaptive,  hidden_layer:  (500, 250, 500),  activation: tanh |

Table A9. MLP Final Results – Month

| **Training Set (Percentage)** | **Testing Set (Percentage)** | **Precision** | **Recall** | **F1-Score** | **Accuracy** | **Parameter Setting**  **(Optimal)** |
| --- | --- | --- | --- | --- | --- | --- |
| 90% | 10% | 0.93 | 0.93 | 0.93 | 0.93 | solver: lbfgs,  max_iter: 400,  learning_rate: constant ,  hidden_layer:  (500, 250, 500),  activation: relu |
| 80% | 20% | 0.92 | 0.92 | 0.92 | 0.92 | solver: lbfgs,  max_iter: 200,  learning_rate: constant,  hidden_layer:  (500, 250, 500),  activation: tanh |
| 70% | 30% | 0.93 | 0.93 | 0.93 | 0.93 | solver: lbfgs,  max_iter: 900,  learning_rate: adaptive,  hidden_layer:  (500, 500),  activation: tanh |
| 60% | 40% | 0.93 | 0.93 | 0.93 | 0.93 | solver: sgd,  max_iter: 600,  learning_rate: adaptive,  hidden_layer:  (100, 50, 100),  activation: tanh |
| 50% | 50% | 0.94 | 0.94 | 0.94 | 0.94 | solver: lbfgs,  max_iter: 800,  learning_rate: constant,  hidden_layer:  (100, 100),  activation: relu |

Table A10. MLP Final Results – Percentage

| **Training Set (Month)** | **Testing Set (Month)** | **Precision** | **Recall** | **F1-Score** | **Accuracy** | **Parameter Setting**  **(Optimal)** |
| --- | --- | --- | --- | --- | --- | --- |
| 7, 8 | 9 | 0.94 | 0.94 | 0.94 | 0.94 | embed_dim: 64,  ff_dim: 96,  num_heads: 2,  dropout: 0.1,  activation: 'tanh',  optimizer: 'adam',  learning_rate: 0.01 |
| 7, 9 | 8 | 0.94 | 0.94 | 0.94 | 0.94 | embed_dim: 96,  ff_dim: 96,  num_heads: 2,  dropout: 0.3,  activation: 'tanh',  optimizer: 'adam',  learning_rate: 0.01 |
| 8, 9 | 7 | 0.94 | 0.94 | 0.94 | 0.94 | embed_dim: 240,  ff_dim: 96,  num_heads: 2,  dropout: 0.2,  activation: 'tanh',  optimizer: 'adam',  learning_rate: 0.01 |

Table A12. Keras Final Results – Month

| **Training Set (Percentage)** | **Testing Set (Percentage)** | **Precision** | **Recall** | **F1-Score** | **Accuracy** | **Parameter Setting**  **(Optimal)** |
| --- | --- | --- | --- | --- | --- | --- |
| 90% | 10% | 0.96 | 0.96 | 0.96 | 0.96 | embed_dim: 96,  ff_dim: 256,  num_heads: 2,  dropout: 0.2,  activation: 'relu',  optimizer: 'adam',  learning_rate: 0.01 |
| 80% | 20% | 0.94 | 0.94 | 0.94 | 0.94 | embed_dim: 126,  ff_dim: 256,  num_heads: 2,  dropout: 0.1,  activation: 'relu',  optimizer: 'adam',  learning_rate: 0.01 |
| 70% | 30% | 0.94 | 0.94 | 0.94 | 0.94 | embed_dim: 160,  ff_dim: 128,  num_heads: 2,  dropout: 0.0,  activation: 'tanh',  optimizer: 'adam',  learning_rate: 0.01 |
| 60% | 40% | 0.92 | 0.92 | 0.92 | 0.92 | embed_dim: 126,  ff_dim: 256,  num_heads: 2,  dropout: 0,  activation: 'tanh',  optimizer: 'adam',  learning_rate: 0.01 |
| 50% | 50% | 0.92 | 0.95 | 0.92 | 0.92 | embed_dim: 126,  ff_dim: 256,  num_heads: 3,  dropout: 0.1,  activation: 'tanh',  optimizer: 'adam',  learning_rate: 0.01 |

Table A13. Keras Final Results – Percentage

| **Training Set (Month)** | **Testing Set (Month)** | **Random**  **Forest** | **SVM** | **AdaBoost** | **GradientBoost** | **Stacking**  **Model** | **Voting**  **Model** |
| --- | --- | --- | --- | --- | --- | --- | --- |
| 7, 8 | 9 | 0.96 | 0.95 | 0.95 | 0.91 | 0.97 | 0.94 |
| 7, 9 | 8 | 0.96 | 0.93 | 0.95 | 0.92 | 0.97 | 0.94 |
| 8, 9 | 7 | 0.95 | 0.93 | 0.94 | 0.92 | 0.97 | 0.94 |

Table A13. Ensemble Model Final Results (F1-Score) – Month

| **Training Set (Percentage)** | **Testing Set (Percentage)** | **RandomForest** | **SVM** | **AdaBoost** | **GradientBoost** | **Stacking**  **Model** | **Voting**  **Model** |
| --- | --- | --- | --- | --- | --- | --- | --- |
| 90% | 10% | 0.96 | 0.95 | 0.95 | 0.92 | 0.97 | 0.95 |
| 80% | 20% | 0.94 | 0.94 | 0.95 | 0.92 | 0.97 | 0.94 |
| 70% | 30% | 0.96 | 0.95 | 0.94 | 0.92 | 0.97 | 0.94 |
| 60% | 40% | 0.96 | 0.94 | 0.94 | 0.92 | 0.97 | 0.94 |
| 50% | 50% | 0.96 | 0.93 | 0.93 | 0.92 | 0.97 | 0.94 |

Table A14. Ensemble Model Final Results (F1-Score) – Percentage

| **Training Set (Month)** | **Testing Set (Month)** | **Naive Bayes Classifier** | **SVM** | **Random Forest** | **XGBoost** | **Ensemble - Stacking** | **MLP** |
| --- | --- | --- | --- | --- | --- | --- | --- |
| 7, 8 | 9 | 0.90 | 0.95 | 0.94 | 0.94 | 0.93 | 0.95 |
| 7, 9 | 8 | 0.84 | 0.94 | 0.91 | 0.90 | 0.94 | 0.93 |
| 8, 9 | 7 | 0.91 | 0.94 | 0.94 | 0.93 | 0.93 | 0.92 |

Table A15. Model Evaluation Results (F1-Score) - Month

| **Training Set (Percentage)** | **Testing Set (Percentage)** | **Naive Bayes Classifier** | **SVM** | **Random Forest** | **XGBoost** | **Ensemble - Stacking** | **MLP** |
| --- | --- | --- | --- | --- | --- | --- | --- |
| 90% | 10% | 0.88 | 0.95 | 0.93 | 0.91 | 0.94 | 0.95 |
| 80% | 20% | 0.88 | 0.94 | 0.93 | 0.91 | 0.95 | 0.93 |
| 70% | 30% | 0.88 | 0.94 | 0.94 | 0.92 | 0.95 | 0.94 |
| 60% | 40% | 0.87 | 0.94 | 0.92 | 0.92 | 0.93 | 0.93 |
| 50% | 50% | 0.88 | 0.95 | 0.92 | 0.91 | 0.93 | 0.94 |

Table A16. Model Evaluation Results (F1-Score) - Percentage
